# The relationship between endothelial progenitor cells and pulmonary arterial hypertension in children with congenital heart disease

**DOI:** 10.1186/s12887-019-1884-x

**Published:** 2019-12-17

**Authors:** Hong-Xiao Sun, Guo-Ju Li, Zhan-Hui Du, Zhen Bing, Zhi-Xian Ji, Gang Luo, Si-Lin Pan

**Affiliations:** 1 Medical College, Qingdao University, Qingdao, 266071 Shandong China; 2 Heart Center, Qingdao Women and Children’s Hospital, Qingdao, 266034 Shandong China

**Keywords:** Child, Congenital heart disease, endothelial progenitor cells, pulmonary arterial hypertension

## Abstract

**Background:**

Pulmonary arterial hypertension (PAH) caused by congenital heart disease (CHD) is very common in clinics. Some studies have shown that PAH is related to the number of endothelial progenitor cells (EPCs), but there is no report on the relationship between PAH and the number of EPCs in children with CHD.

**Methods:**

In this study, a total of 173 cases with CHD (from 0 to 6 years old) were collected. According to the mean pulmonary arterial pressure (mPAP) measured by right heart catheterization, these cases were divided into PAH groups (including high PAH group, mPAP> 25 mmHg, *n* = 32, and the middle PAH group, 20 mmHg ≤ mPAP≤25 mmHg, *n* = 30) and non-PAH group (mPAP< 20 mmHg, *n* = 111). Peripheral blood was taken for flow cytometry, and the number of EPCs (CD133+/KDR+ cells) was counted. The number of EPCs /μL of peripheral blood was calculated using the following formula: EPCs /μL = WBC /L × lymphocytes % × EPCs % × 10^− 6^.

**Results:**

The median EPCs of the non-PAH group, middle PAH group and high PAH group is 1.86/μL, 1.30 /μL and 0.98/μL, respectively. The mPAP decreases steadily as the level of EPCs increases (*P* < 0.05). After adjustment of gender, age and BMI, the number of EPCs was significantly associated with a decreased risk of high PAH (OR = 0.37, 95% CI: 0.16–0.87, *P* < 0.05). However, EPCs was not significantly associated with middle PAH (*P* > 0.05).

**Conclusion:**

The findings revealed that the EPCs and high PAH in patients with CHD correlate significantly and EPCs may become an effective treatment for PAH in patients with CHD. EPCs may be a protective factor of high PAH for children with CHD.

## What is known of this subject

- Pulmonary arterial hypertension (PAH) caused by congenital heart disease (CHD) is very common in the clinic, about 4–10% of CHD patients develop PAH, the prevalence of CHD-related PAH is about 25 people per million in the general population; patients with CHD and PAH have at least a doubled mortality risk compared with patients with CHD without PAH.
- To our knowledge, there were few studies of the relationship between endothelial progenitor cells (EPCs) and CHD-related PAH. The purpose of this study was to investigate the relationship between the number of EPCs and CHD-related PAH in children.

## What is new from this study

- This study was the first to study the relationship between EPCs and PAH in children with CHD.
- We found that mPAP declined steadily with the increase of the level of EPCs.
- The EPCs and high PAH in CHD patients correlate significantly.

## Background

Pulmonary arterial hypertension (PAH) is a common clinical disease which can exist independently. It is mainly the manifestation of many diseases when they progress to a certain stage, namely secundum PAH [1]. In clinic, left-to-right shunt congenital heart disease (CHD) will increase the right cardiac blood flow, thus damaging the pulmonary artery and causing PAH [2]. The statistics show that about 4–10% of CHD patients develop PAH [3–7]. The global prevalence of PAH is about 1, and 80% of the patients live in developing countries. The prevalence of CHD-related PAH is about 25 people per million in the general population. The deaths of patients with both PAH and CHD are at least twice as many as those without PAH [1]. PAH is very common in children, mainly due to CHD [2, 7, 8].

In 1997, Asahara et al. [9] first isolated a kind of cells from human peripheral blood that could be cultured into endothelial cells in vitro. Stimulated by various factors, these cells entered the peripheral blood from the bone marrow to participate in the formation of new blood vessels and repair the damaged endothelial cells. These cells were called endothelial progenitor cells (EPCs). EPCs are bone-marrow-derived cells with the ability to differentiate into endothelial cells, repair and regenerate damaged pulmonary blood vessels. Previous experimental studies have found that EPCs can be applied to the treatment of lung injury [10–12]. There have been many studies demonstrating that the number of EPCs is related to PAH, but the results are inconsistent [13–16]. To our knowledge, there are no studies on the relationship between EPCs and PAH in children with CHD. The purpose of this study was to investigate the relationship between the number of EPCs and PAH in children with CHD and to explore the possibility of applying EPCs to treatment of PAH in children with CHD.

## Methods

### Subjects

From August 2011 to September 2017, 173 patients with CHD aging from 0 to 6 years old and hospitalized in Qingdao Women and Children’s Hospital were selected as the study subjects, including 98 having patent ductus arteriosus (PDA), 58 with atrial aeptal defect (ASD) and 17 with ventricular septal defect (VSD). All patients were preoperative. Patients with lung diseases, connective tissue diseases, HIV infection, portal hypertension, schistosomiasis and other diseases that may cause the increase of PAH were excluded in this study.

### Measurements

According to the “2010 ESC Guidelines for the Management of Grown-up Congenital Heart Disease”, the diagnosis of CHD was based on echocardiography [17]. Mean pulmonary arterial pressure (mPAP) was measured by right heart catheterization. According to “the 6th World Symposium on Pulmonary Hypertension” [18], patients were divided into PAH groups, including high PAH group (mPAP> 25 mmHg, 32 patients) and the middle PAH group (20 mmHg ≤ mPAP≤25 mmHg, 30 patients); the non-PAH group (mPAP< 20 mmHg, *n* = 111).

Peripheral blood samples were collected for flow cytometry using a FACS Calibur cytometer (Becton Dickinson, USA). We added FcR-blocking reagent (eBiosciences, USA) into the samples and incubated them, then added selective antibodies (Mouse anti-human CD133-PE, Miltenyi Biotec GmbH, Germany; Mouse anti-human KDR-APC, R&D Systems, USA). Labeled immunoglobulins (Mouse anti-human IgG1-PE, Shenzhen Jingmei, China; Mouse anti-human IgG1-APC, Shenzhen Jingmei, China) were applied for each sample as a negative control. After incubation, the lysing solution (MasterTech, USA) was used to eliminate erythrocytes cells. Horizontal centrifuge (Shanghai anting, China), centrifuged at 250 g at 4°C with brake for 5 min to remove the supernatant. Finally, the number of CD133+/KDR+ cells was counted. The number of monocytes in human peripheral blood is about 0.30–0.80 × 10^9^ /L; the proportion of EPCs is about 0.01%; the number of EPCs is estimated to be 0.30–1.00 /μL [19, 20]. Thus, the number of EPCs < 1.00 /μL was used as the control. The number of EPCs /μL of peripheral blood was calculated by using the following formula: EPCs/μL = WBC/L × lymphocytes% × EPCs% × 10^–^ 6[9]. Body mass index (BMI) was calculated as the subject’s weight (kg) divided by the height squared (meters).

### Statistical analysis

The data were expressed as the mean ± standard deviation for normally distributed continuous variables and compared by one-way analyses of variance. Median values (inter-quartile range) were used and compared by Mann–Whitney U test when the parameters exhibited skewed deviations. The number and proportions in each subgroup of categorical variables were compared by the Mann–Whitney U test. The multiple comparisons in each subgroup were made by Bonferroni test or Student-Newman-Keuls test. A multinomial logit analysis was performed to explore relationships between the number of EPCs and PAH severity after adjustment of age, gender, and BMI. All *P*-values less than 0.05 were considered to be statistically significant. All of the analyses were performed using SAS 9.4 (SAS Institute Inc., Cary, NC, USA).

## Results

In the present study, a total of 173 CHD patients met all analysis criteria, including 111 (64.16%) non-PAH patients, 30 (17.34%) middle PAH patients and 32 (18.50%) high PAH patients. The general characteristics of the non-PAH group, middle PAH group and high PAH group are shown in Table 1. The median age of the non-PAH patients, middle PAH patients and high PAH patients is 2 years, 2 years, and 0 years, respectively. The median age was lower in the high PAH group than in the middle PAH and non-PAH groups (Student-Newman-Keuls test, all *P* < 0.05). BMI, WBC, and lymphocyte are similar in the three subgroups (all *P* > 0.05). The proportion of EPCs> 1.00/μL of the non-PH group, middle PH group and high PH group is 70.27, 70.00 and 50.00, respectively, and the proportion of EPCs > 1.00/μL is significantly reduced among the high PAH patients compared with the EPCs<1.00/μL(*P* = 0.05). With the non-PAH group as a reference, the associations between EPCs and mPAP levels were estimated in multinomial logistic models (Table 2). EPCs was significantly associated with a decreased risk of high PAH (OR = 0.42, 95%CI: 0.19–0.95, *P* < 0.05). We next adjusted age and gender, and examined the associations between the number of EPCs and mPAP levels, and the results showed that the number of EPCs was significantly associated with a decreased risk of high PAH (OR = 0.38, 95%CI: 0.17–0.87, *P* < 0.05). After adjustment of gender, age and BMI, the EPCs was also significantly associated with a decreased risk of high PAH (OR = 0.37, 95%CI: 0.16–0.87, *P* < 0.05). However, EPCs was not significantly associated with middle PAH (*P* > 0.05).

**Table 1:** Demographic characteristic of the study population

Variables	PAH			Non-PAH	statistics	P value
	20-25 mmHg	> 25 mmHg	Total (> 20 mmHg)			
Number	30	32	62	111		
Female, %	20 (66.67)	20 (62.50)	40 (64.52)	55 (49.55)	2.64	0.10
Age, mean (SD), years	2 (1–3)	0 (0–2) ^bc^	1 (0–3)	2 (0–3)	6.53	0.04
BMI, mean (SD), kg/m^2^	16.09 ± 2.24	15.17 ± 2.04	15.62 ± 2.18	16.16 ± 2.47	2.23	0.11
WBC(×10^9^/L)	8.50 (7.30–10.16)	8.80 (7.47–9.96)	8.75 (7.40–10.00)	8.70 (6.80–10.20)	0.44	0.80
lymphocyte(×10^9^/L)	4.46 (3.51–5.45)	5.07 (3.53–6.51)	4.97 (3.51–6.20)	4.69 (3.37–6.19)	0.43	0.81
EPCs> 1.00 /μL, %	21 (70.00)	16 (50.00)	37 (59.68)	78 (70.27)	3.75	0.05
mPAP, mmHg	22.00 ± 1.72^a^	35.69 ± 8.76^bc^	29.06 ± 9.38	14.82 ± 2.04	−11.80	**< 0.0001**

a: denotes statistical significance between Non-PAH and PAH (20-25 mmHg);

b: denotes statistical significance between Non-PAH and PAH (> 25 mmHg);

c: denotes statistical significance between PAH (20-25 mmHg) and PAH (> 25 mmHg)

**Table 2:** Association between EPCs and PAH by multivariate logistic regression models

EPCs, μL Levels	PAH (20-25 mmHg) Odds Ratio(95%CI)			PAH(> 25 mmHg) Odds Ratio(95%CI)		
	Unadjusted	Age and gender adjusted	Multivariate adjusted+	Unadjusted	Age and gender adjusted	Multivariate adjusted+
≤1.00	1	1	1	1	1	1
> 1.00	0.99 (0.41–2.38)	0.95(0.39–2.31)	0.95(0.39–2.33)	0.42(0.19–0.95) *	0.38(0.17–0.87) *	0.37(0.16–0.87) *

*Compared with EPCs≤1.00: *P* < 0.05; +Adjusted: gender, age, and BMI

## Discussion

PAH is a pathophysiological syndrome caused by known or unknown factors, characterized by pulmonary vascular contraction, remodeling and in situ thrombosis. Generally, endothelial dysfunction, oxidative stress, inflammation, and angiogenesis disorders are considered as the main causes. The progressive increase of pulmonary vascular resistance restricts blood flow and causes an abnormal increase of pulmonary artery pressure, eventually leading to hemodynamic changes in pulmonary circulation, impaired right heart function, and even death [2, 21, 22]. The previous PAH is defined as mPAP ≥25 mmHg in children > 3 months of age at sea level, mPAP ≥25 mmHg, PCWP < 15 mmHg, PVR index > 3 WU × m2 [23]. According to the guideline, the normal mPA*P* value is 14 ± 3.30 mmHg when people are resting, and the mPAP should not exceed 20 mmHg under normal circumstances even considering age, gender, race, and other related factors. In our study, the mPAP is 14.82 ± 2.04 mmHg in non-PAH groups, similar to the above study. Recent studies have found that patients with mPAP ranging 20 mmHg < mPAP≤25 mmHg have a significantly increased risk of disease progression. Therefore, 20 mmHg < mPAP≤25 mmHg can be considered as the early stage of pulmonary vascular disease [18, 24].

Some studies have suggested that peripheral blood EPCs may be derived from bone marrow EPCs, and blood sampling is a relatively non-invasive method compared with bone marrow puncture [25, 26]. Therefore, studies on EPCs have shifted from bone marrow EPCs to peripheral blood EPCs in recent years. It can be seen that EPCs in peripheral blood are biomarkers of various pathophysiological states. Because flow cytometry is relatively sensitive to the identification of biomarkers, it has high universality and usability. During the past decade, this technology has developed rapidly and become the mainstream method for separation, classification, and analysis of EPCs [27–29]. EPCs are counted by flow cytometry using different markers or combinations of them. There is still a considerable controversy over the phenotype of EPCs. Currently, the common expression of CD34, CD133, and VEGFR2 is most widely used for the identification of EPCs. The human expression of VEGFR2 is also known as KDR [16, 19, 24]. CD133 is expressed in early EPCs but absent in mature endothelial cells, which is the surface marker of EPCs [30, 31]. KDR is an important marker of endothelial tissue [32, 33]. However, CD34 expressed in endothelial cells at any stage cannot be used as a specific marker of EPCs [12, 27, 33]. Therefore, flow cytometry was used in this study to define CD133+/KDR+ cells as EPCs. It has been proved that CD133+/KDR+ cells can differentiate into endothelial cells in vitro and in vivo, contributing to the re-endothelialization of the left heart, and promoting endothelial regeneration at the site of ischemia and vascular injury [12]. Since there is no unified definition and classification of EPCs surface markers at present, some researchers have used combinations of other surface markers to identify different EPCs subgroups [19, 20, 34].

In the present study, both unadjusted and adjusted mPAP decline steadily with the increase in the level of EPCs. For patients with EPCs> 1.00/μL, the risk of high PAH (> 25 mmHg) was significantly lower than that for patients with EPCs< 1.00/μL (*P* < 0.05), regardless of adjusting gender, age, and BMI or not. However, a significant difference in risk of PAH between EPCs and the middle PAH (20-25 mmHg) was not found (*P* > 0.05). PAH severity is negatively correlated with the number of EPCs, suggesting that the reduction of EPCs increases the risk of PAH among patients with CHD. At present, there are no studies on the relationship between EPCs and PAH in children with CHD. In the study by Zhu et al., [1, 14] the number of EPCs is reduced in idiopathic PAH, which is consistent with our study. Liu et al. [15] also found that EPCs in PAH combined with the chronic obstructive pulmonary disease was decreasing. However, Schiavon et al. [13] concluded that elevated EPCs in patients with end-stage PAH may be associated with a long course of illness, leading to a compensatory proliferation of EPCs. Other surface markers have been used to identify other EPCs subsets. Some scholars [16, 35]. argue that reduced EPCs levels may lead to endothelial dysfunction in CHD patients, triggering PAH. Due to the continuous damage of pulmonary artery endothelial cells caused by PAH, EPCs are mobilized to repair them, and then EPCs are gradually exhausted. The higher the mPAP is, the more EPCs are consumed, while EPCs cells are reduced, thereby affecting the repair of pulmonary artery endothelial cells and causing a vicious cycle. Sen et al. [12] believe EPCs to be an important marker of cardiovascular diseases.

Through this study, we believe that in patients with CHD, endothelial cells may be damaged or functioned due to the increase of blood flow or accelerated flow rate in the pulmonary artery and EPCs are largely used to repair endothelial cells, leading to a decrease in the number. EPCs can be assumed to be a protective factor of PAH and associated with PAP. According to the present study, no difference in the middle PAH group is probably because that the condition of these patients is relatively mild, and the peripheral blood EPCs are enough to repair the endothelial injury; so the EPCs are not excessively consumed. Recent studies have found that EPCs play an important role in maintaining vascular homeostasis, reversing pulmonary vascular remodeling and promoting angiogenesis; their function is to participate in the differentiation of vascular smooth muscle cells and potential cardiomyocytes by releasing cytokines, growth factors, and chemokines [34, 36, 37]. In addition, various cytokines and VEGF may inhibit the mobilization of bone marrow EPCs and indirectly reduce peripheral EPCs [38, 39]. By releasing angiogenic factors, anti-apoptotic factors and anti-inflammatory factors, some cells can be differentiated or have paracrine to play the therapeutic role of progenitor cells [10, 16]. Thus, EPCs have been used in treating PAH in animal studies and achieved good results [40–42]. Lavoie et al. [43] applied EPCs in the experimental treatment of idiopathic PAH patients and found that the PAP decreased to varying degrees, but could not be completely reduced to a normal level. Hence, the treatment should still be combined with pulmonary artery antihypertensive drugs. It has provided a good prospect for the radical treatment of PAH.

According to our study, EPCs may be a protective factor of PAH in children with CHD. Therefore, EPCs may be an effective treatment for patients with CHD complicated with PAH, if the PAP cannot be reduced to a normal level after surgery,.

Strengths and limitation: There are few studies about the relationship between EPCs and PAH in children with CHD. A rigorous experimental design was carried out strictly. Three types of CHD (PDA, ASD, and VSD) are included in the study, which may affect the final results. We will continue to conduct research on a specific CHD to obtain more scientific results. Due to the low incidence of PAH in children with CHD, the sample size of the experiment was small which may affect the results. In the further experiment, we will collect more samples to confirm our conclusion.

## Conclusion

The current study revealed the relationship between EPCs and PAH in children with CHD. The findings suggest that a reduction of the number of EPCs in children with CHD may be associated with an increase in mPAP. There was a significant correlation between EPCs and high PAH in CHD patients and EPCs may become an effective treatment for PAH in CHD patients.

## Data Availability

The datasets used and/or analysed during the current study are available from the corresponding author on reasonable request.

## Abbreviations

CHD: Congenital Heart Disease
EPCs: Endothelial Progenitor Cells
mPAP: Mean Pulmonary Arterial Pressure
PAH: Pulmonary Arterial Hypertension
